# Early timepoint ^99m^Tc-DPD whole body scintigraphy and quantitative SPECT/CT imaging for diagnosis of cardiac ATTR amyloidosis

**DOI:** 10.1186/s13550-026-01466-0

**Published:** 2026-06-12

**Authors:** Stephan Settelmeier, Lukas Kessler, Noah Hammersen, Maria Papathanasiou, Christoph Rischpler, Alexander Carpinteiro, Sara Oubari, Lars Michel, Julia Vogel, Tim Wollenweber, Jing Ning, Marcus Hacker, Clemens P. Spielvogel, Zohreh Varasteh, Wolfgang P. Fendler, Francesco Barbato, Pedro Fragoso Costa, Stephan Leyser, Tienush Rassaf, Ken Herrmann, Tugce Telli, David Kersting

**Affiliations:** 1 Department of Cardiology and Vascular Medicine, West German Heart and Vascular Center, University Hospital Essen, Hufelandstrasse 55, 45147 Essen, Germany; 2 West German Amyloidosis Center, University Hospital Essen, Hufelandstr. 55, 45147 Essen, Germany; 3 Department of Nuclear Medicine, University Hospital Essen, Hufelandstrasse 55, Essen, 45147 Germany; 4 Department of Diagnostic and Interventional Radiology and Neuroradiology, University Hospital Essen, Hufelandstrasse 55, 45147 Essen, Germany; 5 Department of Cardiology and Vascular Medicine, University Hospital Frankfurt, Goethe University, Frankfurt, Germany; 6 German Center for Cardiovascular Research (DZHK), Partner Site Rhein-Main, 60596 Frankfurt, Germany; 7 Department of Nuclear Medicine, Klinikum Stuttgart, Kriegsbergstr. 60, 70174 Stuttgart, Germany; 8 Department of Hematology and Stem Cell Transplantation, University Hospital Essen, Hufelandstrasse 55, 45147 Essen, Germany; 9 Department of Biomedical Imaging and Image-Guided Therapy, Division of Nuclear Medicine, Medical University of Vienna, Spitalgasse 23, Vienna, 1090 Austria

**Keywords:** Cardiac amyloidosis, ATTR, DPD-SPECT, Nuclear cardiology, Perugini score

## Abstract

**Background:**

Cardiac transthyretin amyloidosis (ATTR-CM) is a progressive myocardial disease ultimately leading to heart failure. Standard diagnostic workup includes ^99m^Tc-DPD scintigraphy performed after 2.5–3 h. The purpose of this study is to compare early (1 h after injection) to late imaging of ^99m^Tc-DPD scintigraphy and SPECT for the detection of ATTR-CM. Early imaging could improve patient comfort and examination efficiency.

**Results:**

50 patients undergoing ^99m^Tc-DPD scintigraphy and SPECT for suspected ATTR-CM were included. Imaging was performed at both 1 h and 2.5–3 h post-injection. Tracer uptake was assessed visually (Perugini score), semi-quantitatively (e.g., heart-to-mediastinum ratio, HMR), and quantitatively (e.g., maximum standardized uptake value, SUV_max_). ATTR-CM was diagnosed in 28% of patients. Median visual Perugini score was significantly higher for early imaging (0.5 vs. 0, *p* < 0.001). Findings were validated using an external validation cohort. Intraclass correlation coefficients between early and late imaging were very good. Visual evaluation of early imaging demonstrated comparable sensitivity, specificity, and overall diagnostic accuracy to late imaging. HMR and SUV_max_ from early imaging showed higher diagnostic accuracy. No false-negative results were observed. Visual evaluation using the Perugini score was slightly less consistent between readers in early compared to late imaging, with lower inter-observer agreement at 1 h.

**Conclusions:**

Early ^99m^Tc-DPD imaging provides diagnostic performance equivalent to conventional late imaging while substantially enhancing patient comfort and workflow efficiency. Adoption of early imaging protocols, complemented by quantitative and semi-quantitative analysis, may streamline ATTR-CM diagnostics, enabling timely diagnosis and treatment.

**Graphical abstract:**

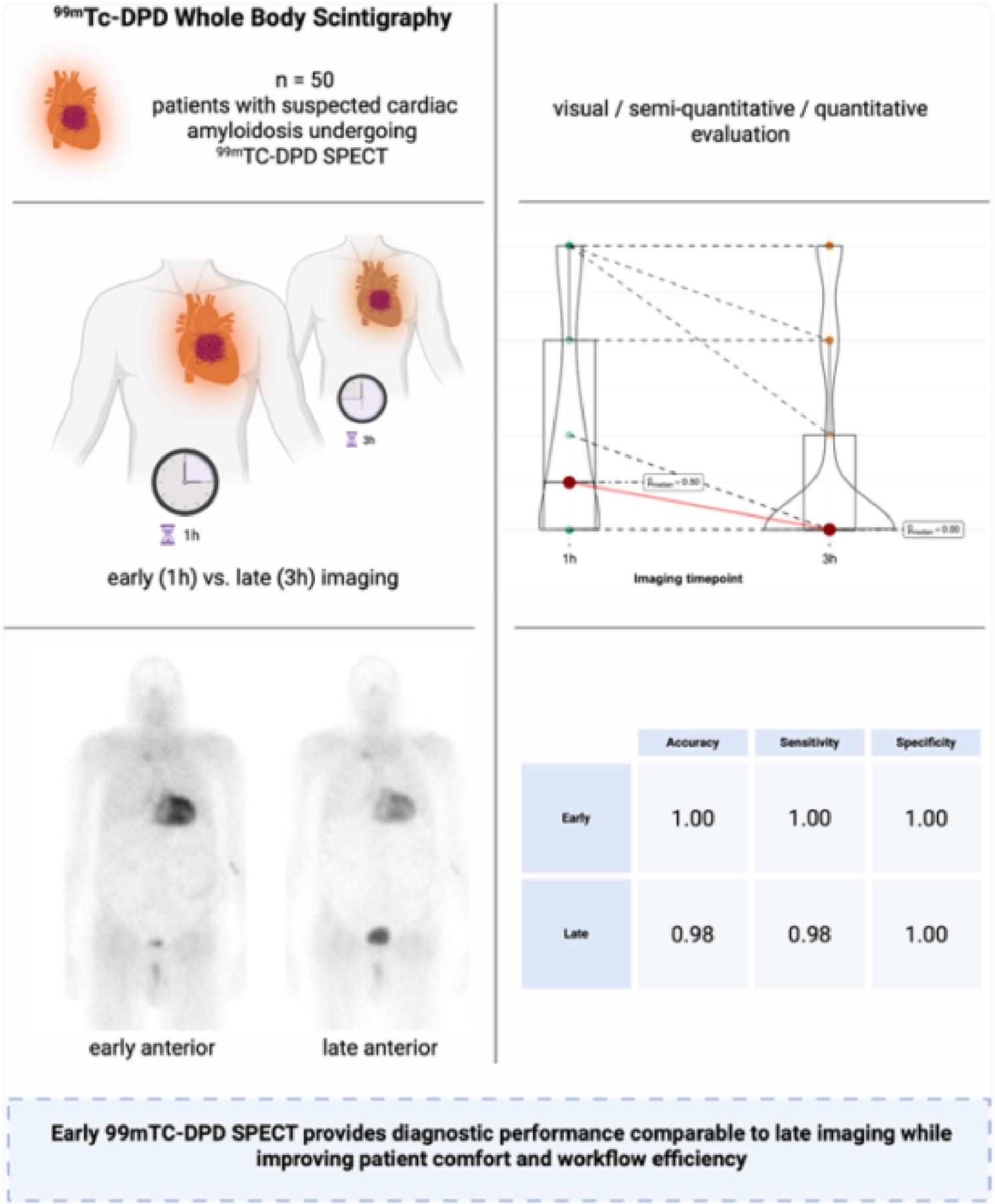

## Introduction

Cardiac amyloidosis is a progressive myocardial disease with increasing incidence that ultimately leads to amyloid cardiomyopathy (CM), an important cause of heart failure [1, 2]. Increased awareness of the disease is highlighted in the recent guidelines for the diagnosis and therapy of CM [3]. The two main subtypes of CM are immunoglobulin light chain amyloidosis (AL) and transthyretin amyloidosis (ATTR), which can be hereditary (hATTR) or wild-type (wtATTR) [4].

ATTR-CM and AL-CM differ in epidemiology, affection of additional organ systems, mean survival, and treatment options [5–8]. While therapeutic options for AL-CM include chemotherapy of the underlying disease (e.g., monoclonal gammopathy or multiple myeloma) [4], treatment of ATTR-CM was, for a long time, limited to supportive measures [6]. This changed with the approval of TTR stabilizer therapy and several other treatment options that are available for clinical use or in advanced clinical development [9]. ATTR-CM has a high prevalence and poor prognosis, with a median survival of only 3–5 years [7]. Recently approved treatment options have been shown to reduce all-cause mortality and cardiovascular hospitalizations, slow functional decline, and improve quality of life [6].

Endomyocardial biopsy remains the gold standard for diagnosing ATTR-CM [2, 9] but is an invasive procedure. Nuclear medicine imaging, including planar scintigraphy or cardiac single photon emission computed tomography (SPECT) using bone-avid tracers [10–14] or positron emission tomography (PET) using amyloid-specific tracers [15–19], are non-invasive techniques that can alternatively be performed to detect cardiac amyloid burden with high sensitivity and specificity.

Planar scintigraphy imaging and additional SPECT are simple and well-established procedures that have found their way into routine clinical practice for diagnosis of ATTR-CM [20]. Moreover, identification of additionally affected organ systems is possible [21, 22]. In Europe, ^99m^Tc-diphosphono-propanodicarboxylic acid (^99m^Tc-DPD) or ^99m^Tc-hydroxymethylene-diphosphonate acid (^99m^Tc-HDP) are used as tracers for ATTR-CM detection [10, 21, 23]. In the US, these tracers are typically not available and instead, ^99m^Tc-pyrophosphate (^99m^Tc-PYP) is used for imaging of ATTR-CM [14, 24]. All agents allow for diagnosis of ATTR-CM by visual evaluation, e.g., using the Perugini score [10, 11, 25], and while differentiation between ATTR-CM and AL-CM by qualitative/semi-quantitative comparisons of the cardiac tracer uptake in comparison to reference regions or quantitative SPECT imaging is more challenging [10, 12, 14, 23].

Using ^99m^Tc-PYP, imaging is typically performed one hour after tracer injection [14], whereas using ^99m^Tc-DPD, the common interval between injection and imaging is 2–3 h [10]. A shorter interval of one hour increases patient comfort, allows more efficient scheduling, and facilitates timely diagnosis. This is of importance to meet the increasing demand for examinations.

The aim of this study was to compare early (one hour after injection) versus late (2.5–3 h after injection) ^99m^Tc-DPD scintigraphy for detection of ATTR-CM. Furthermore, for both time points, (semi-)quantitative metrics from planar scintigraphy and SPECT imaging are additionally compared.

## Methods

### Study population & ethics approval

50 consecutive patients who were referred to our center for suspected ATTR-CM and subsequently received a confirmed diagnosis between February 2022 and February 2024 and willing to participate were included in this retrospective observational study. Referral and allocation were carried out by our cardiology and hematology specialties. Observational study was approved by the local institutional ethics board (Medical Faculty of the University of Duisburg-Essen, 21-10243 BO) and written informed consent was requested.

### Diagnosis of cardiac amyloidosis

A review of clinical health records was performed for diagnosis of CM; this diagnosis was used as gold standard in the following evaluations. Diagnosis of ATTR-CM was validated by positive endomyocardial biopsy or on an imaging basis by fulfillment of the widely clinically accepted Gillmore criteria [11] requiring a positive bone scan (at least Perugini score 2) and absence of pointers of plasma cell disease.

### Image acquisition

Injection of ^99m^Tc-DPD was performed after written informed consent for clinical examination. Scintigraphy and thoracic SPECT images were acquired 1 h and 2.5-3 h after tracer injection according to clinical protocol. CT images were used for attenuation correction and quantitative image reconstruction [26]. Images were acquired with either a Symbia Intevo or a Symbia T2 SPECT/CT system (both Siemens Medical Solutions, Erlangen, Germany).

### Image evaluation

Different visual, quantitative, and semi-quantitative image evaluation metrics were applied:

(i) Visual evaluation of planar images: Cardiac tracer uptake on planar scintigraphy images was visually evaluated using the Perugini score [10]:

- Grade 0: No cardiac uptake.
- Grade 1: Mild cardiac uptake, less than bone uptake.
- Grade 2: Moderate cardiac uptake, similar to bone uptake.
- Grade 3: Intense cardiac uptake, greater than bone uptake.

(ii) Semi-quantitative evaluation of planar images: Cardiac tracer uptake on planar scintigraphy images was semi-quantitatively evaluated by comparing count rates in a cardiac-centered region-of-interest (ROI) to count rates in specific reference region ROIs. For this purpose, the heart-to-contralateral lung ratio (HLR) from anterior whole body planar images was calculated, comparing the mean counts per pixel in the cardiac ROI to those in a contralateral lung ROI. Similarly, the heart-to-mediastinum ratio (HMR) was calculated, comparing the mean counts per pixel in the cardiac ROI to those in a mediastinal reference ROI.

(iii) Quantitative evaluation of SPECT images: Cardiac tracer uptake on quantitative SPECT images was assessed by determining the maximum standardized-uptake-value (SUV_max_) in a cardiac-centered volume-of-interest (VOI). Quantitative image reconstruction and image evaluation were performed using a previously described method established at our center [26]. In brief, the whole heart was segmented and the SUV_max_ value in the cardiac VOI was determined.

(iv) Semi-quantitative evaluation of SPECT images: For semi-quantitative evaluation of cardiac tracer uptake on SPECT images, data were normalized by blood pool and bone uptake [26]. To estimate blood pool and bone uptake, VOIs of 1 cm^3^ volume were placed in a thoracal vertebral bone (that did not show any signs of degenerative changes) and the descending thoracal aorta [26]. The SUV_max_ values in these VOIs were used to calculate the heart-to-vertebra ratio (HVR) and the heart-to-blood pool ratio (HBR) using the following definitions [26]:

- Heart-to-vertebra ratio (HVR): SUV_max cardiac_ / SUV_max bone_.
- Heart-to-blood pool ratio (HBR): SUV_max cardiac_ / SUV_max blood pool_.

(V) Inter-observer validity.

For the analysis of inter-observer validity, the Perugini scores of the early and late imaging time points were evaluated independently by a total of 3 experienced board-certified nuclear medicine physicians in a blinded manner. In case of discrepant results, majority consensus read was obtained.

### Validation

19 patients (male/female = 14 (74%)/5 (26%), mean age 78.1 years) with wtATTR-CM were scanned at the Medical University of Vienna at an early (1 h) and late (3 h) time point using a SPECT/CT system (Symbia Intevo, Siemens Medical Solutions AG, Erlangen Germany). Image analysis was performed using Hermes Hybrid 3D (Hermes Medical Solutions, Stockholm, Sweden). Perugini scores were determined according to clinical routine. Observational study was approved by the local institutional ethics board (EK769/2010). Each individual gave written informed consent prior to imaging. Findings were compared to the Essen cohort.

### Statistical analysis

Statistical tests applied included the Wilcoxon signed-rank test to compare the visual Perugini grade between early and late images, intraclass correlation coefficient (ICC; two-way, mixed model) to compare semiquantitative and quantitative metrics between early and late images, and receiver operating characteristic (ROC) analysis to assess diagnostic accuracy (applying Youden’s J statistic to derive optimal cut-off values). Differences in various semi-quantitative parameters between the ATTR-CM and non-CM groups were evaluated using the Mann-Whitney U test. For the ICC, additionally 95% confidence intervals (CI) were reported. For all tests, P-values (P) < 0.05 were considered statistically significant. Fleiss’ kappa was calculated for evaluation of inter-observer validity including 95% confidence intervals. Statistical analysis was performed using R statistical software in version 4.5.1 (R Foundation for Statistical Computing, Vienna, Austria).

## Results

### Patient characteristics

**Fig. 1:**
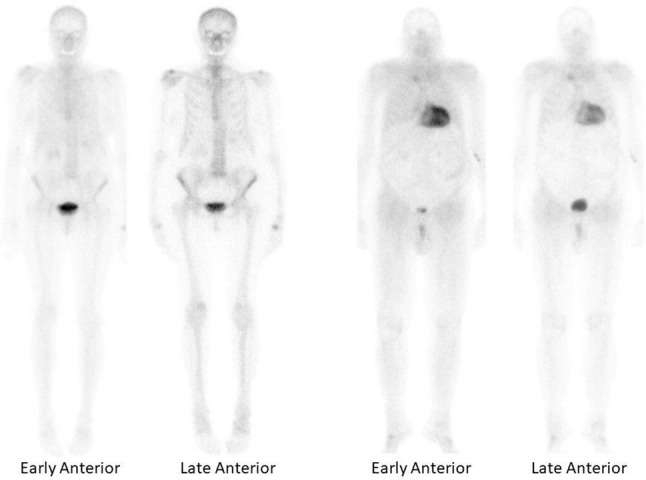
Image examples. Planar scintigraphy images (early and late) from a patient without (left) and from a patient with ATTR-CM (right)

A total of 50 patients were included. Of these, 14 (28.0%) presented with ATTR-CM. Patient characteristics are shown in Table 1. Image examples of patients with and without ATTR-CM (at early and late imaging time points) are shown in Fig. 1.

**Table 1:** Patient characteristics

**Characteristics**	
*Demographics*	
Male/Female	34/16
Age (y) (mean ± SD)	78 ± 9
Weight (kg) (mean ± SD)	81.5 ± 17.9
Administered Activity (MBq) (mean ± SD)	648.7 ± 107.0
*Perugini score (late time point)*	
0	36 (72%)
1	2 (4%)
2	5 (10%)
3	7 (14%)
*Perugini score (early time point)*	
0	25 (50%)
1	11 (22%)
2	3 (6%)
3	11 (22%)
*Diagnosis*	
No ATTR-CM	36 (72 %)
ATTR-CM	14 (28 %)
**NHYA Functional Class**	
0-I	5 (10.0%)
II	14 (28.0%)
III	29 (58.0%)
IV	2 (4.0%)

### Perugini score

**Fig. 2:**
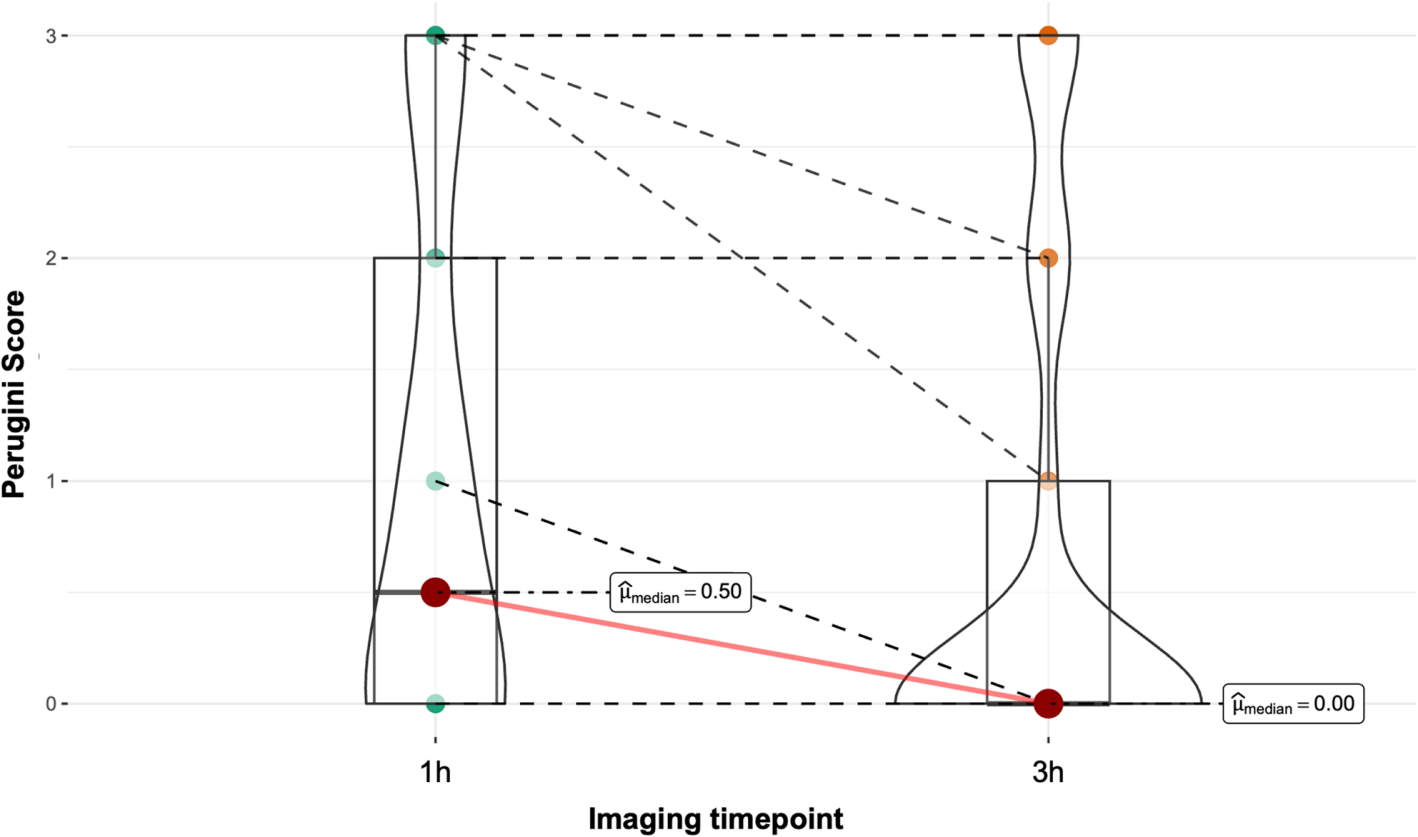
Wilcoxon signed-rank test. Visualization of Wilcoxon signed-rank test results comparing ordinal Perugini scores from early (1 h; green) and late (3 h; orange) imaging. Dashed lines connect paired scores from early to late imaging, illustrating within-patient changes; for example, patients graded as Perugini 2 showed no change between time points. Violin plots depict the distribution of scores, with embedded boxplots indicating the interquartile range. Large red circles mark the median score at each time point; the solid red line shows median change between early and late imaging

**Fig. 3:**
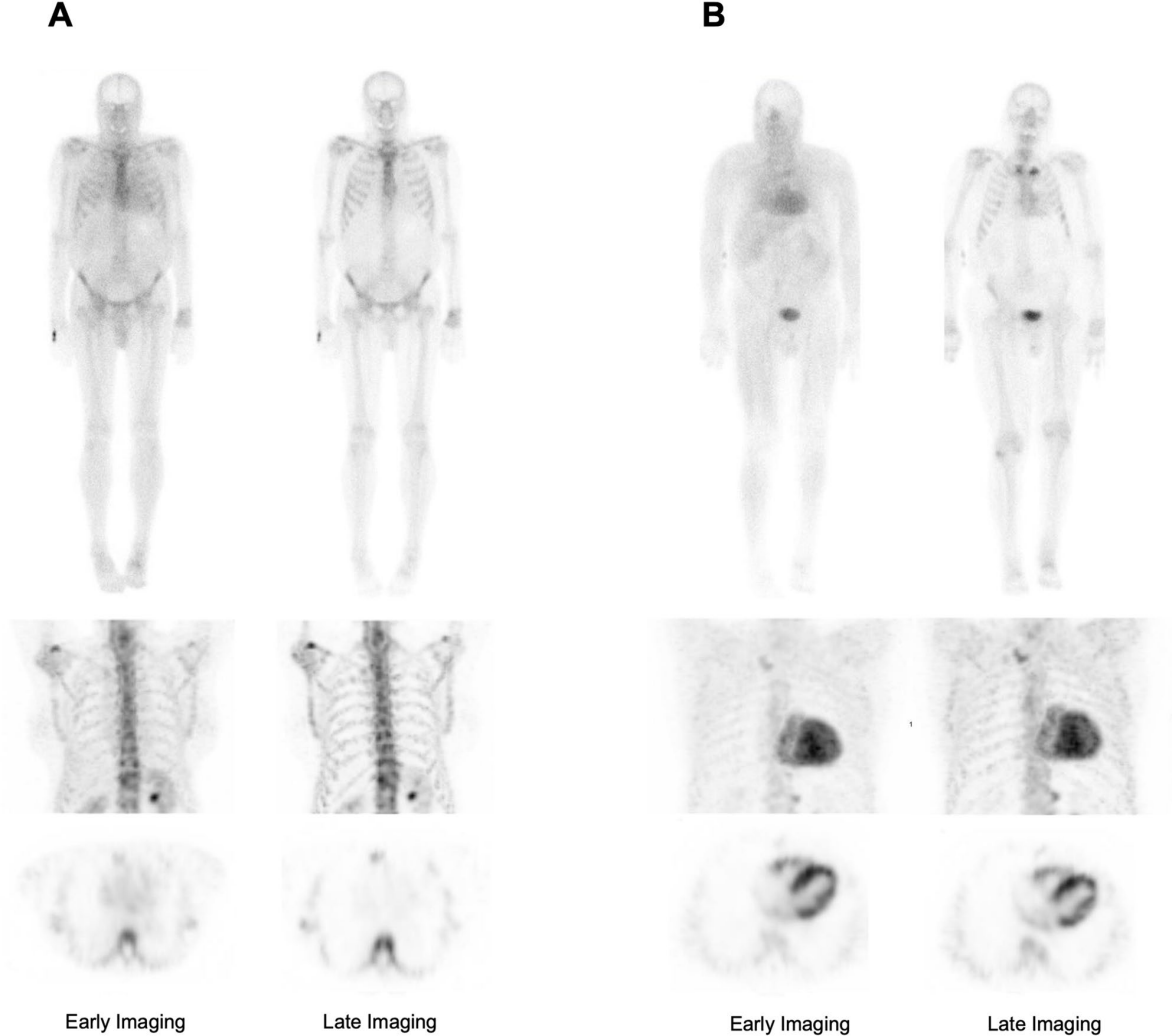
Example of shifting Perugini scores between early and late imaging Planar scintigraphy and SPECT images (early and late) from one patient at time points 1 h and 3 h. Perugini score is shifting from 1 to 0 (**A**) and 3 to 1 (**B**), respectively

Median visual Perugini score was significantly higher for early imaging (0.5 vs. 0, *p* < 0.001). Detailed evaluation revealed a shift from Perugini 0 in late imaging to Perugini 1 in early imaging in 11 patients (22%). Two patients (4%) showed a shift from Perugini 1 in late imaging to Perugini 3 in early imaging. Both patients were diagnosed with biopsy-proven ATTR-CM (Fig. 2). Example images for shifted Perugini scores are displayed in Fig. 3. Moreover, two patients (4%) showed a shift in Perugini score from 2 in late to 3 in early imaging.

### Quantitative and semi-quantitative uptake parameters

All (semi-)quantitative parameters derived from early and late planar and SPECT images were significantly higher in patients with ATTR-CM compared to patients without ATTR-CM (Table 2), indicating the potential of these metrics to differentiate patients with ATTR-CM.

**Table 2:** Different semi-quantitative parameters from planar and SPECT/CT images

Parameter	No ATTR-CM	ATTR-CM	*P* value
**Early imaging (median (IQR))**			
HMR	0.7 (0.6–0.8)	2.0 (1.4–2.6)	< 0.001
HLR	1.3 (1.2–1.4)	2.3 (1.7–2.8)	< 0.001
Cardiac SUV_max_	3.7 (3.0-4.1)	14.9 (12.9–18.8)	< 0.001
HBR	1.5 (1.4–2.1)	6.7 (4.5–10.5)	< 0.001
HVR	0.6 (0.4–0.7)	2.8 (2.3–3.4)	< 0.001
**Late imaging (median (IQR))**			
HMR	0.4 (0.3–0.5)	1.4 (1.0-2.1)	< 0.001
HLR	1.0 (0.9–1.1)	1.8 (1.6–2.6)	< 0.001
Cardiac SUV_max_	2.8 (2.1–3.9)	11.7 (9.4–17.2)	< 0.001
HBR	1.8 (1.4–3.8)	4.8 (4.0-10.1)	< 0.001
HVR	0.4 (0.2–0.6)	1.7 (1.3–2.1)	< 0.001

CM = cardiac amyloidosis, ATTR = transthyretin, IQR = interquartile range, HMR = heart-to-mediastinum ratio; HLR = heart-to-contralateral-lung ratio, SUV = standardized uptake value, HBR = heart-to-blood-pool ratio, HVR = heart-to-vertebra ratio

Good to excellent correlation was observed between late and early time points with higher correlations for (semi-)quantitative parameters. ICC for Perugini grading was 0.85 (*p* < 0.001). ICC for HMR, HLR, SUV_max_, HBR, HVR were 0.90 (*p* < 0.05), 0.89 (*p* < 0.001), 0.90 (*p* < 0.001), 0.79 (*p* < 0.001), 0.78 (*p* < 0.001), respectively.

### Sensitivity, specificity, accuracy, and AUC for diagnosis of ATTR-CM

All parameters showed high accuracy for diagnosing ATTR-CM (accuracy reaching from 0.80 to 1.0). The best performing metrics derived from planar scintigraphy and SPECT imaging were: Perugini score (late and early time point), HMR (early time point), SUV_max_ (early time point), HCR (early time point). Detailed results, indicating also AUC, sensitivity, specificity, positive predictive value (PPV) and negative predictive value (NPV) are summarized in Table 3.

**Table 3:** Different cut-off values derived from various semiquantitative analyses and their detection accuracy

Parameter	Cut-off	Youden Index	Accuracy	Sensitivity	Specificity	AUC	PPV	NPV
**Early imaging**								
**HMR**	0.98	1.00	1.00	1.00	1.00	1.00	1.00	1.00
**HLR**	1.54	0.86	0.90	1.00	0.86	0.97	0.74	1.00
**Cardiac SUV**_**max**_	8.55	1.00	1.00	1.00	1.00	1.00	1.00	1.00
**HBR**	3.82	0.90	0.96	0.93	0.97	0.96	0.93	0.97
**HVR**	1.25	1.00	1.00	1.00	1.00	1.00	1.00	1.00
**Late imaging**								
**HMR**	0.85	0.93	0.98	0.93	1.00	0.97	1.00	0.97
**HLR**	1.35	0.80	0.92	0.86	0.94	0.92	0.86	0.94
**Cardiac SUV**_**max**_	7.05	0.94	0.96	1.00	0.94	0.99	0.87	1.00
**HBR**	3.96	0.63	0.80	0.86	0.78	0.87	0.60	0.93
**HVR**	0.78	0.86	0.90	1.00	0.86	0.97	0.74	1.00

HMR = heart-to-mediastinum ratio; HLR = heart-to-contralateral-lung ratio, SUV = standardized uptake value, HBR = heart-to-blood-pool ratio, HVR = heart-to-vertebra ratio

### Inter-observer variability in late and early imaging of Perugini score

Early time point studies showed a higher inter-observer variability compared to late time point imaging (Fleiss’ kappa late: 0.82 (95% CI: 0.71–0.94), early: 0.58 (95% CI: 0.47–0.68). At early imaging, the blood pool activity remains relatively high compared to the 3-h time point (mean ± SD SUV_max blood-pool_ 2.3 ± 0.98 vs. 1.73 ± 0.94), whereas bone activity increases from the early to the late time point (SUV_max bone_ 0.43 ± 1.02 vs. 0.58 ± 1.07).

### External validation

In the external validation cohort, the median visual grade was significantly higher at early imaging (3 vs. 2, *p* < 0.001). Detailed evaluation revealed a predominant downgrade from Perugini 3 at 1 h to Perugini 2 at late imaging in 10 patients (52.6%). The remaining patients had no change. No upward shifts were observed in this cohort (Table 4).

**Table 4:** External validation cohort

Parameter	Early imaging	Late imaging
Age (years), mean ± SD	78.1 ± 7.6	78.1 ± 7.6
Sex (M/F)	14 (74%) / 5 (26%)	14 (74%) / 5 (26%)
n	19	19
Perugini (mean)	2.79	2.26
Perugini (minimum)	2	2
Perugini (maximum)	3	3
Perugini 0, n (%)	0 (0%)	0 (0%)
Perugini 1, n (%)	0 (0%)	0 (0%)
Perugini 2, n (%)	4 (21%)	14 (74%)
Perugini 3, n (%)	15 (79%)	5 (26%)
Shift early → late, n (%)	Downgrade by 1: 10 (53%)	Unchanged: 9 (47%); Upgrade: 0

## Discussion

The study demonstrated an overall good agreement between early and late imaging for various cardiac uptake parameters. Visual interpretation (Perugini score) and (semi-)quantitative uptake values show up to perfect separation and accuracy for diagnosing ATTR-CM in the investigated patient cohort at both time points and no substantial difference between early and late imaging was observed (Table 3). This was also reflected by up to excellent intraclass correlation coefficients between both imaging time points.

Our results indicate that early imaging can be as reliable as late imaging for diagnosing CM. The high accuracy, sensitivity, and specificity further support the feasibility of early imaging in clinical practice. Detailed analysis of Perugini score between early and late imaging showed systematically higher grades observed in early images (Fig. 2). Most change shifts were from Perugini 0 (late) to Perugini 1 (early) or from Perugini 2 (late) to Perugini 3 (early) (Fig. 2). These changes do not alter diagnosis of ATTR-CM but should be considered when interpreting early images, particularly if visual assessment alone is used: Perugini 1 cases were more abundant in early imaging (Table 1). Because cardiac uptake at 1 h post-injection is systematically more pronounced than at 3 h, established thresholds derived from late imaging – most importantly Perugini ≥ 2 for non-biopsy diagnosis of ATTR-CM in line with the Gillmore criteria – should not be transferred uncritically to the early time point. In the present cohort the same dichotomous threshold preserved diagnostic accuracy at both time points, but Perugini 1 was clearly more frequent at 1 h and should currently be regarded as an indication for additional late imaging rather than as positive evidence of disease. The higher residual blood-pool signal at early imaging likely reduces myocardial-to-background contrast (HBR 3.1 ± 6.3 vs. 3.7 ± 6.5), which may explain the decreased inter-observer variability in Perugini score interpretation. More specifically, residual blood-pool activity at 1 h reduces the visual contrast between the myocardium and surrounding background, while the still-rising bone uptake by the late time point provides a clearer reference against which the relative cardiac signal can be judged. As a consequence, mild cardiac uptake (Perugini 1) is more difficult to differentiate from absent uptake (Perugini 0) at 1 h than at 3 h, and a slightly higher proportion of equivocal interpretations is to be expected. In clinical practice this should be considered when relying on visual scoring alone; complementary use of (semi-)quantitative parameters – which in our cohort showed excellent inter-method agreement (ICC ≥ 0.78) and high diagnostic accuracy at 1 h – can support the visual read, particularly in borderline cases.

Only in two cases, Perugini scores switched from 3 in early imaging to 1 in late imaging. This led to the need for invasive diagnostics and in both patients ATTR-CM was proven by biopsy. Importantly, overall, the observed switches in Perugini grades had no impact on clinical diagnosis in this study, because no false-negative and false-positive result were observed. However, these results are yet limited by the sample size and the low number of Perugini 1 cases at late imaging which might require special attention.

Because of the good accuracy of the early imaging in differentiation between positive or negative diagnosis, most of the patients could be diagnosed at an early imaging time point. Due to shifts from 0 (late imaging) to 1 (early imaging) special attention must be paid to these patients. A practical approach can be to image only patients presenting with Perugini score 1 additionally at the later time point to reduce unnecessary invasive diagnostics. It has to be noted that, most likely due to the different blood pool activity, the inter-observer variability was higher in the early imaging group without affecting diagnostic accuracy.

The systematic elevation of early scores is likely attributable to tracer kinetics: At 1 h post-injection blood pool and myocardial activity are higher and later decreasing, whereas bone activity shows slower kinetics and is still increasing from the 1 h to the 3 h imaging time point. This behavior is well reflected by quantitative measurements in our cohort and earlier evaluation of the external validation cohort [27]. As the Perugini score visually compares myocardial to bone uptake, these processes explain higher Perugini grades at early imaging.

Compared with the initial cohort, the external validation results reinforce the observation that early imaging yields higher visual grades. However, the magnitude of change appears greater in the validation cohort, where nearly half of the patients exhibited a one-grade decrease, while the initial cohort showed a smaller proportion of downward shifts. Together, these findings validate the robustness of early imaging as a sensitive time point for detecting tracer uptake and strengthen the reproducibility of this effect across independent patient populations. Performing ^99m^Tc-DPD imaging at an earlier time point significantly enhances patient comfort by reducing the waiting period from 2.5 to 3 h to only 1 h. This benefit is particularly relevant for elderly patients and those with mobility limitations, for whom prolonged waiting can be burdensome. In addition, early imaging increases scheduling flexibility and allows clinics to examine more patients within the same timeframe - an important consideration given the rising demand for ATTR-CM diagnostics as awareness and referral rates continue to grow. In the context of increasing outpatient-based healthcare delivery, particularly in Germany and across the EU, a shorter imaging interval may also facilitate more efficient planning and resource allocation, thereby improving overall workflow in the outpatient sector. Comparable findings have been made for ^99m^Tc-PYP [28, 29]. Imakhanova et al. [28] reported a high concordance between 1 h and 3 h ⁹⁹ᵐTc-PYP planar and SPECT/CT imaging in patients with suspected cardiac amyloidosis, with comparable sensitivity and specificity at both time points and a clear indication that the early protocol is sufficient for routine differentiation of ATTR-CM. Masri et al. [29] similarly demonstrated, in a larger clinical cohort, that an efficient 1 h ⁹⁹ᵐTc-PYP imaging protocol provides reliable diagnostic information for transthyretin cardiac amyloidosis without compromising visual or H/CL ratio-based interpretation. Together, these studies support the feasibility of early imaging for ⁹⁹ᵐTc-PYP; our results extend this concept to ⁹⁹ᵐTc-DPD and confirm that the systematic upward shift in early visual grades, attributable to the differing kinetics of myocardial and bone uptake, does not impair overall diagnostic accuracy when combined with (semi-)quantitative parameters. One important advantage of the proposed approach is the substantial reduction in waiting time for approximately three-quarters of patients, as only about one-quarter would need to undergo delayed imaging. We acknowledge that this workflow adjustment may require organizational changes, particularly in the outpatient setting. However, we are confident that the improved schedulability for the majority of patients, with only a one-hour waiting period, would considerably ease the operational burden and enable a more predictable utilization of the gamma camera, even if the total number of examinations may slightly increase overall. Formal evaluation of the operational impact should be undertaken before the routine adoption of this targeted delayed-imaging approach. In the present cohort, only 4 of 50 patients (8%) presented with a Perugini score of 1 at the early time point, indicating that the additional delayed scan would be required in only a small minority of patients in routine practice. The proposed two-step workflow nevertheless requires prospective validation – ideally with a higher proportion of borderline cases – and a formal evaluation of its operational impact before broad clinical adoption.

In addition, early imaging may offer technical advantages: With the physical half-life of ^99m^Tc-DPD being 6 h, roughly 20% higher residual activity is expected at 1-hour post-injection. Thus, either a lower injected dose or a shorter acquisition time could be applied without compromising overall image quality. As myocardium shows an earlier peak uptake than bone tissue, there is an optimal time window after injection in which myocardial uptake has already reached a plateau, whereas bone uptake has not yet peaked—maximizing the contrast between heart and bone. This observation supports the notion that the Perugini score may require adjustment for early images, as the heart-to-bone ratio differs from that seen at later time points and further underscores the importance of integrating (semi-)quantitative parameters [30]. These considerations are supported by phantom measurements [31].

Beyond clinical routine, the results of this study may also facilitate international multicenter trials. Early imaging protocols combined with (semi-)quantitative approaches could provide standardized, reproducible imaging results across centers and regions, independent of tracer availability. The high diagnostic accuracy of these parameters emphasizes their potential for user-independent and harmonized assessment of ATTR-CM.

### Limitations

A limitation of this study is the relatively small sample size in both cohorts, which, despite receiving data from two centers, restricts the generalizability of the findings. In addition, patient recruitment was performed within the context of clinical routine, which inherently introduces heterogeneity in referral patterns and diagnostic work-up. This also resulted in a comparatively low proportion of patients with confirmed ATTR-CM, which could limit the statistical power for subgroup analyses. Furthermore, only few borderline cases were included, limiting the evidence in patients with borderline results in Perugini scores.

### Clinical perspective

This study demonstrates that early ^99m^Tc-DPD scintigraphy offers reliable diagnostic accuracy comparable to late imaging. A practical approach can be to image only patients presenting with Perugini score 1 additionally at the later time point to reduce unnecessary invasive diagnostics. By reducing waiting time and improving scheduling capacity, early imaging protocols can optimize patient-centred care and support standardized, efficient diagnostic pathways for cardiac amyloidosis.

## Conclusion

This study demonstrates that early ^99m^Tc-DPD scintigraphy provides diagnostic reliability comparable to conventional late imaging. The most immediate clinical benefit lies in improved patient comfort, particularly for elderly patients and those with mobility limitations, by reducing the waiting period from 2.5 to 3 h to 1 h. Confirmation in larger prospective cohorts with a higher proportion of borderline cases is warranted before early imaging can be broadly recommended for routine clinical practice.

## Data Availability

The datasets generated during and/or analyzed during the current study are available from the corresponding author on reasonable request.

## Abbreviations

CM: Amyloid cardiomyopathy
ATTR-CM: Transthyretin cardiomyopathy
DPD – ^99m^Tc: Diphosphono-propanodicarboxylic acid
SPECT: Single photon emission computed tomography
SUV_max_: Maximum ctandardized uptake value
HMR: Heart-to-mediastinum ratio
HLR: Heart-to-lung ratio
HVR: Heart-to-vertebra ratio
HBR: Heart-to-blood pool ratio
ICC: Intraclass correlation coefficient
